# Comparative Genomic Analysis of a Clinical Isolate of *Klebsiella quasipneumoniae* subsp. *similipneumoniae*, a KPC-2 and OKP-B-6 Beta-Lactamases Producer Harboring Two Drug-Resistance Plasmids from Southeast Brazil

**DOI:** 10.3389/fmicb.2018.00220

**Published:** 2018-02-16

**Authors:** Marisa F. Nicolás, Pablo Ivan Pereira Ramos, Fabíola Marques de Carvalho, Dhian R. A. Camargo, Carlene de Fátima Morais Alves, Guilherme Loss de Morais, Luiz G. P. Almeida, Rangel C. Souza, Luciane P. Ciapina, Ana C. P. Vicente, Roney S. Coimbra, Ana T. Ribeiro de Vasconcelos

**Affiliations:** ^1^Laboratório Nacional de Computação Científica, Petrópolis, Brazil; ^2^Instituto Gonçalo Moniz, Fundação Oswaldo Cruz, Salvador, Brazil; ^3^Fundação Ezequiel Dias, Belo Horizonte, Brazil; ^4^Laboratório de Genética Molecular de Microrganismos, Instituto Oswaldo Cruz, Fundação Oswaldo Cruz, Rio de Janeiro, Brazil; ^5^Neurogenômica, Fiocruz Institute Renê Rachou, Belo Horizonte, Brazil

**Keywords:** *Klebsiella quasipneumoniae* subsp *similipneumoniae*, KPC-2, OKP-B-6, silver resistance, nosocomial infection, complete genome sequence

## Abstract

The aim of this study was to unravel the genetic determinants responsible for multidrug (including carbapenems) resistance and virulence in a clinical isolate of *Klebsiella quasipneumoniae* subsp. *similipneumoniae* by whole-genome sequencing and comparative analyses. Eighty-three clinical isolates initially identified as carbapenem-resistant *K. pneumoniae* were collected from nosocomial infections in southeast Brazil. After RAPD screening, the KPC-142 isolate, showing the most divergent DNA pattern, was selected for complete genome sequencing in an Illumina HiSeq 2500 instrument. Reads were assembled into scaffolds, gaps between scaffolds were resolved by *in silico* gap filling and extensive bioinformatics analyses were performed, using multiple comparative analysis tools and databases. Genome sequencing allowed to correct the classification of the KPC-142 isolate as *K. quasipneumoniae* subsp. *similipneumoniae*. To the best of our knowledge this is the first complete genome reported to date of a clinical isolate of this subspecies harboring both class A beta-lactamases KPC-2 and OKP-B-6 from South America. KPC-142 has one 5.2 Mbp chromosome (57.8% G+C) and two plasmids: 190 Kbp *p*KQPS142a (50.7% G+C) and 11 Kbp *p*KQPS142b (57.3% G+C). The 3 Kbp region in *p*KQPS142b containing the *bla*_KPC−2_ was found highly similar to that of *p*Kp13d of *K. pneumoniae* Kp13 isolated in Southern Brazil in 2009, suggesting the horizontal transfer of this resistance gene between different species of *Klebsiella*. KPC-142 additionally harbors an integrative conjugative element ICE*Pm1* that could be involved in the mobilization of *p*KQPS142b and determinants of resistance to other classes of antimicrobials, including aminoglycoside and silver. We present the completely assembled genome sequence of a clinical isolate of *K. quasipneumoniae* subsp. *similipneumoniae*, a KPC-2 and OKP-B-6 beta-lactamases producer and discuss the most relevant genomic features of this important resistant pathogen in comparison to several strains belonging to *K. quasipneumoniae* subsp. *similipneumoniae* (phylogroup II-B), *K. quasipneumoniae* subsp. *quasipneumoniae* (phylogroup II-A), *K. pneumoniae* (phylogroup I), and *K. variicola* (phylogroup III). Our study contributes to the description of the characteristics of a novel *K. quasipneumoniae* subsp. *similipneumoniae* strain circulating in South America that currently represent a serious potential risk for nosocomial settings.

## Introduction

The prevalence of isolation of carbapenem-resistant *Klebsiella pneumoniae* strains in nosocomial infections is increasing, posing a serious therapeutic problem given the limited number of effective antimicrobial agents. Recently in the USA, isolates belonging to the related *K. pneumoniae* species, namely *K. variicola*, and *K. quasipneumoniae*, were isolated and shown to harbor capsular synthesis K type locus (KL) KL19 and KL1, respectively, and both carried the *Klebsiella pneumoniae* carbapenemase (KPC) gene (Long et al., 2017). Also, contrary to what was previously thought, *K. quasipneumoniae* and *K. variicola* strains can be as virulent as extended-spectrum beta-lactamases (ESBL)-producing *K. pneumoniae* strains, causing invasive infections, and mortality at rates statistically similar to those of *K. pneumoniae* strains (Long et al., 2017), which confers resistance to carbapenem antibiotics. Even more recently, a hypervirulent *Klebsiella quasipneumoniae* subsp. *similipneumoniae* isolated from a patient with chronic liver disease in India that belongs to novel sequence type ST2320 and possesses the K1 capsular serotype has been reported (Shankar et al., 2017). These findings accentuate the concern for the potential spread of multidrug resistance and increased virulence capacity among various *Klebsiella* species.

Because of overlapping biochemical profiles, phenotypic tests are unable to differentiate between *K. pneumoniae, K. quasipneumoniae*, or *K. variicola* (Alves et al., 2006; Bowers et al., 2016), and this limitation may lead to underreporting infections caused by the latter species. Notably, the capsular serotypes and MLST types among *K. quasipneumoniae* and *K. variicola* strains recovered from human infections are diverse and novel K-types and MLST types are being found in isolates of these two species (Brisse et al., 2013; Garza-Ramos et al., 2015; Long et al., 2017). Core chromosomal beta-lactamases have been proposed as molecular markers to differentiate *Klebsiella* species, i.e., *K. pneumoniae* (SHV restricted), *K. quasipneumoniae* (OKP restricted), and *K. variicola* (LEN restricted) (Haeggman et al., 2004; Fonseca et al., 2017). However, this method has some complications, since there are some SHV beta-lactamase genes encoded on plasmids and *K. variicola* isolates carrying chromosomal OKP-B instead of LEN (Long et al., 2017). Nevertheless, when genomic sequences are available, the average nucleotide identity (ANI) is a robust proxy for genomic relatedness between strains, and has been used to differentiate *Klebsiella* species with BLASTn (cut-off 96% identity) (Brisse et al., 2014). Thus, a better understanding of the genomics aspects of *K. quasipneumoniae* and *K. variicola* strains will help toward the development of improved diagnostics.

In 2011 we collected an isolate from a nosocomial infection in Southeast Brazil, initially identified as a KPC-producing *K. pneumoniae* (KPC-142) by clinical routine methods. In this report, by determining its complete genome sequence, we show that this is an isolate of KPC-producing *K. quasipneumoniae* subsp. *similipneumoniae* (KPC-Kqps) instead of KPC-producing *K. pneumoniae* (KPC-Kp). Genomic analyses showed that KPC-142 harbors a *cps* cluster related to KL16 type, an integrative conjugative element termed ICE*Pm1* and a novel combination of known MLST alleles. Besides *bla*_KPC−2_ it carries additional antibiotic resistance genes, including *bla*_OKP−B−6_, *aph*(3')-VIa for an aminoglycoside-modifying enzyme, silver resistance genes, and possess a significant amount of virulence factors. By a genomic comparison between KPC-142 and strains belonging to other *Klebsiella* phylogroups we highlight the most relevant genomic features, including the N-acetyl-neuraminic acid catabolism pathway that appears only in *K. quasipneumoniae* subsp. *similipneumoniae* strains and which is known to play a role in the bacterial pathogenesis.

## Material and methods

### Bacterial strains used in this study

From 83 KPC isolates carrying the *bla*_KPC_ gene collected from nosocomial infections in Minas Gerais state (Southeast Brazil) in 2011 we selected the isolate named KPC-142, which displayed the most divergent DNA pattern by RAPD cluster analysis (Figure S1). This isolate was initially assigned as *K. pneumoniae* KPC-142 and susceptibility tests were performed according to CLSI guidelines 2017, using Vitek 2 system and AST-N239 cards (bioMérieux, Inc., Durham, NC) according to the manufacturer's instructions and using Gram-negative identification (GN ID) cards (Bobenchik et al., 2017).

The bacterial isolate was grown overnight on nutrient agar and the DNA was extracted using the method originally described by Coimbra et al. (1999). The isolate KPC-142 is maintained in the certified strains collection of Ezequiel Dias Foundation (FUNED), Belo Horizonte, Brazil.

For the comparative genomic analyses and determination of average nucleotide identity (ANI), we used a bacterial strain panel representative of each *Klebsiella* phylogroup (Holt et al., 2015) that included: (i) nine *K. quasipneumoniae* subsp. *similipneumoniae* (phylogroup II-B) (HKUOPA4, HKUOPJ4, HKUOPL4, KPC-142, ATCC 700603, 07A044 T, MGH 44, 193_KOXY, and 385_ECLO), (ii) nine *K. quasipneumoniae* subsp. *quasipneumoniae* (phylogroup II-A) (UCICRE14, 01A030 T, FI HV 2014, MGH96, 18A069, ARLG-2711, PO1285, AK_SD_007, and 21_GR_13), nine *K. pneumoniae* (phylogroup I) (DSM 30104 T, Kp13, 1084, CAV1193, HS11286, KCTC 2242, KP617, MGH 78578, and NTUH-K2044), and nine *K. variicola* (phylogroup III) (BZ19, GJ1, UCICRE10, MGH 40, MGH 20, UCI 18, BIDMC 61, BIDMC88, and MGH 80), totaling 36 bacterial genomes. An additional 31 isolates were included for phylogenetic studies (see section MLST classification and phylogeny).

### Genome sequencing, assembly, annotation, and bioinformatics analyses

Genomic DNA was sequenced on an Illumina HiSeq 2500 sequencer using Nextera XT paired-end run with a 500-bp insert library at the High-Throughput Sequencing Platform of the Oswaldo Cruz Foundation (Fiocruz, Rio de Janeiro, Brazil). The assembly of reads into scaffolds, based on 3,804,017 reads, was accomplished using a combination of Newbler v 2.6 (Roche Inc.) and SPAdes 3.10.0 (Bankevich et al., 2012) programs. 99.99% of the bases on the assembled genome had an average Phred quality of >40, calculated by Newbler. Gaps intra- and inter-scaffolds were resolved using a three-phase approach: (1) by extracting consensus sequences that did not form gaps in one of the assemblies; (2) by performing local assemblies of reads falling in gap termini; and (3) using the GapFiller tool (Boetzer and Pirovano, 2012). Further details of each step are presented in the Figure S2.

Genome annotation was performed using the System for Automated Bacterial Integrated Annotation (SABIA) (Almeida et al., 2004). The steps performed by this platform consist of an initial gene prediction process using Glimmer (Delcher et al., 1999) and GeneMark (Besemer et al., 2001) including a start codon correction routine based in the multiple alignment of similar proteins identified by BLASTP against NCBI proteins database (www.ncbi.nlm.nih.gov/protein). CDSs identified after this process were annotated applying an automated annotation pipeline, where each open reading frame (ORF) is submitted to similarity searches using both nucleotide and amino acid sequences by Basic Local Alignment Search Tool (BLAST) (Altschul et al., 1990) against KEGG (www.genome.jp/kegg), NCBI-nr (www.ncbi.nlm.nih.gov/protein) and UniProtKB/Swiss-Prot (www.uniprot.org) databases, in addition to the prediction of protein domains and important catalytic sites using InterPro database (Finn et al., 2017), and the results are made available on the screen for the assessment of expert users. In this process, only CDSs with predicted lengths over 50 aa and at least one BLASTP hit against any of the four databases mentioned above were considered. The transfer RNAs (tRNAs) were detected by tRNAscan-SE (Lowe and Eddy, 1997) and the annotation of ribosomal RNA genes was carried out by RNAmmer (Lagesen et al., 2007).

Further bioinformatics analyses included BLAST searches against AtlasT4SS database (Souza et al., 2012)/Resfinder database (Zankari et al., 2012)/ARDB-Antibiotic Resistance Genes Database (Liu and Pop, 2009)/Comprehensive Antibiotic Resistance Database (Jia et al., 2017) and Virulence Factors database (VFDB) (Chen et al., 2005), which were performed for identification of type 4 secretion systems (T4SS), acquired antimicrobial resistance genes and bacterial virulence factors, respectively. Typing of plasmids was carried out by *in silico* detection using PlasmidFinder database (Carattoli et al., 2014).

Average nucleotide identity (ANI) was calculated using BLASTn (ANIb) in JSpeciesWS (Richter et al., 2016). Reference type strains of *K. quasipneumoniae* belonging to subspecies *K. quasipneumoniae* subsp. *quasipneumoniae* 01A030 (GenBank accession no. GCA_000751755) and *K. quasipneumoniae* subsp. *similipneumoniae* 07A044 (GenBank accession no. GCA_000613225) and type strain *K. pneumoniae* subsp. *pneumoniae* DSM 30104 (GenBank accession no. GCA_000281755), in addition to strain *K. variicola* GJ1 (GenBank accession no. GCA_001989495.1), were used to perform all-versus-all comparisons with a bacterial strain panel including KPC-142. An ANI threshold of ≥96% or greater was considered to delineate species boundaries as it correlates well to DNA-DNA hybridization studies (Goris et al., 2007; Federhen et al., 2016).

Comparative genomic and gene cluster analyses were performed using the Gview tools (Petkau et al., 2010) and Proteinortho (Lechner et al., 2011), respectively. The comparisons involved the bacterial strain panel. In Proteinortho, co-orthologous clustering was calculated using BLASTp, with an identity/coverage cutoff of >90% and *E*-values < 10^−5^. BLASTn Atlas was generated with Gview, applying as parameters identity >70% and E-values < 10^−5^ (Petkau et al., 2010).

The *bla*_KPC−2_-harboring 10,951 bp plasmid of KPC-142 (*p*KQPS142b) was compared with plasmids of *K. pneumoniae* strain Kp13 (*p*Kp13d), *K. pneumoniae* strain A60136 (*p*60136), and *E. coli* (IncQ *p*RSF1010) by BLASTn. The genomic context of the highly similar genes to *p*KQPS142b were obtained using the Gview program (Petkau et al., 2010), with manual editing.

Kaptive tool (Wyres et al., 2016) was used for classification of capsular loci (*cps* cluster) and *wzi* and *wzc* allele typing was performed by querying the predicted nucleotides sequences of both genes against the Institut Pasteur MLST database [bigsdb.pasteur.fr/klebsiella/].

### MLST classification and phylogeny

The allele sequences for seven housekeeping genes (*gapA, infB, mdh, pgi, phoE, rpoB*, and *tonB*) and sequence types (STs) were assigned by using the MLST (http://bigsdb.pasteur.fr/klebsiella/klebsiella.html) and NCBI (www.ncbi.nlm.nih.gov) databases.

In order to investigate the phylogenetic placement of KPC-142, the seven housekeeping genes were concatenated for each of the 36 genomes used in a bacterial strain panel, in addition to *K. quasipneumoniae* subsp. *quasipneumoniae, K. quasipneumoniae* subsp. *similipneumoniae*, and *K. pneumoniae* strains, which sequence typing were closer to that identified to KPC-142. *K. variicola* was used as outgroup. The sequences were aligned in MAFFT using the accurate LINSI strategy (Katoh and Standley, 2013). Poorly aligned regions were removed in the final alignment by Gblocks program (Castresana, 2000), allowing positions with a gap in less than 50% of the sequences. Twenty-four percent of the 12,219 original base positions were retained in the final alignment.

Maximum likelihood tree for the matrix consisting of the seven concatenated loci was constructed with PhyML (Guindon et al., 2009), comprising 67 nucleotide sequences. jModelTest v. 2.1.10 (Darriba et al., 2012) was used to select the best-fit model of nucleotide substitution according to the corrected Akaike information criterion measure. The evolutionary history was inferred using GTR+I+G as nucleotide substitution model, which was chosen as best-scoring in the earlier step, with 1,000 replicates of a nonparametric bootstrap as clade support. Tree editing and annotation were performed using interactive Tree of Life (iTOL) (Letunic and Bork, 2016). A phylogenetic network using the same aligned sequences as input was inferred using the NeighborNet algorithm and untransformed distances (uncorrected-*p*) in SplitsTree4 v. 4.14.6 (Huson and Bryant, 2006). The pairwise homoplasy index (phi) (Bruen et al., 2006) was calculated within SplitsTree4 to evaluate if the recombination events were statistically significant.

### Nucleotide sequence accession number

The Bioproject accession number for KPC-142 is PRJNA383559 and the complete sequences of the chromosome and plasmids *p*KQPS142a and *p*KQPS142b have been deposited in GenBank/NCBI under accession numbers CP023478, CP023479, and CP023480, respectively.

## Results and discussion

### Main features of the chromosome and plasmids sequences of isolate KPC-142

We have sequenced to closure the genome of *K. quasipneumoniae* subsp. *similipneumoniae* KPC-142. This isolate was chosen for sequencing following a RAPD analysis of a panel of multiple *bla*_*KPC*−2_-producing *Klebsiella* clinical isolates (Figure S1), in which KPC-142 presented a very divergent DNA pattern. The KPC-142 genome consists of one 5,217,996 bp chromosome with 57.84% G+C content (4,856 protein-coding genes) and two plasmids, namely *p*KQPS142a with 189,707 bp and 50.71% G+C content (199 protein-coding genes) and *p*KQPS142b with 10,951 bp and 57.32% G+C content (12 protein-coding genes). Also, KPC-142 contains eight copies of 16S rRNAs and 23S rRNA and nine copies of 5S rRNA.

A panel of 35 representative *Klebsiella* isolates belonging to *K. quasipneumoniae, K. pneumoniae* and *K. variicola* was chosen to comparatively investigate the genome of isolate *Kqps*142. The genomic characteristics of all 36 isolates (including *Kqps*142) are shown in Table 1, and the selected strains include the type species of the three *Klebsiella* species compared (and the two *K. quasipneumoniae* subspecies), and cover a wide range of sequence types (STs; including those closer to *Kqps*142), and variable composition of resistance determinants.

**Table 1:** Major genomic characteristics of the panel of *K. quasipneumoniae* subsp. *similipneumoniae* (Kqs), *K. quasipneumoniae* subsp. *quasipneumoniae* (Kqq), *K. pneumoniae* (Kpn), and *K. variicola* (Kv) strains analyzed in this study.

***Klebsiella* species**	**Strain**	**ANI (%)^1^**	**ST**	***wzi***	**KPC-**	**CTX-M-**	**NDM- OXA-**	**SHV-OKP-LEN core**	**SHV- plasmid**	**TEM-**	**NCBI^b^ BioProjet**
Kps	*Kqps*142	99.04	ND	60^*^	KPC-2			OKP-B-6			PRJNA383559^c^
	07A044	100.0	1215	164				OKP-B-1			PRJEB5159^c^
	ATCC700603	99.25	489	171			OXA-2	OKP-B-6	SHV-18		PRJNA307517^c^
	HKUOPA4	99.16	ND	434				OKP-B-6			PRJNA309571^d^
	HKUOPJ4	99.13	ND	434				OKP-B-6			PRJNA309572^d^
	HKUOPL4	99.16	ND	434				OKP-B-6			PRJNA309573^d^
	MGH44	98.95	1435	183				OKP-B-7			PRJNA201959^c^
	193_KOXY	99.05	ND	290				OKP-B-1			PRJNA267549^c^
	385_ECLO	99.15	367	233^a^				OKP-B-6			PRJNA267549^c^
Kqq	01A030^#^	100.0	1528	15				OKP-A-3		TEM-116	PRJEB6037^c^
	UCICRE14	99.02	1437	185				OKP-A-4			PRJNA202000^c^
	FIHV2014	99.10	1807	97				OKP-A-12			PRJNA288524^e^
	MGH96	99.03	2979	425^a^				OKP-A-3			PRJNA271899^c^
	18A069	99.04	1118	165				OKP-A-2			PRJEB5158^c^
	ARLG2711	99.12	2979	17^*^^a^				OKP-A-5			PRJNA339843^c^
	PO1285	98.91	1647	17^*^^a^		CTX-M-15	OXA-1	OKP-A-5		TEM-1	PRJNA351846^c^
	AK_SD_007	98.33	ND	50		CTX-M-15	OXA-1	OKP-A-11			PRJNA351846^c^
	21GR13	98.57	2401	216^*^				OKP-A-11			PRJNA307517^c^
Kpn	DSM30104	100.0	3	3				SHV-1		TEM-1	PRJNA89609^c^
	KP13	99.01	442	154	KPC-2	CTX-M-2	OXA-9	SHV-110	SHV-12	TEM-1	PRJNA78291^c^
	1084	99.08	23	172				SHV-182			PRJNA167369^f^
	CAV1193	99.10	941	25	KPC-2	CTX-M-15	OXA-9	SHV-11	SHV-7	TEM-1	PRJNA246471^c^
	HS11286	99.12	11	74		CTX-M-14		SHV-11		TEM-1	PRJNA78789^c^
	KCTC2242	99.16	375	72				SHV-1			PRJNA67293^g^
	KP617	99.08	ND	2			NDM-1 OXA-232	SHV-28			PRJNA295237^c^
	MGH78578	99.05	38	50				SHV-11	SHV-12	TEM-1	PRJNA31^c^
	NTUHK2044	99.05	23	1				SHV-11			PRJDA21069^f^
Kv	GJ1	100.0	363	53^a^			NDM-9	LEN-2			PRJNA327903^h^
	BZ19	98.96	ND	54				LEN-2			PRJNA238043^c^
	UCICRE10	99.20	596	184^a^				LEN-16			PRJNA201994^c^
	MGH40	99.02	1434	182^a^				LEN-16			PRJNA201955^c^
	MGH20	99.00	1433	32^a^				LEN-2			PRJNA201935^c^
	UCI18	98.90	641	20^a^				LEN-16			PRJNA219268^c^
	BIDMC61	99.22	697	413^a^				LEN-16			PRJNA234149^c^
	BIDMC88	98.82	454	202^a^				LEN-2			PRJNA271899^c^
	MGH80	99.12	ND	66				LEN-16			PRJNA234120^c^

*(1)ANI percent versus the genome used as a reference strain of the species or subspecies. Capsule locus wzi that appear to be variants are indicated with an asterisk*.

(a) *Capsule locus wzi with truncated coding sequence. ICE, integrative conjugative element; ND, not defined*.

(b) Isolate type:

(c) human infection;

(d) mammalian feces;

(e) hypermucoviscous (HV);

(f) liver abscesses;

(g) , 2,3-butanediol-producing Kpn;

(h) *urban river*.

We calculated pairwise average nucleotide identity (ANI) of KPC-142 chromosome sequence including type strains *K. pneumoniae* subsp. *pneumoniae* DSM 30104, *K. quasipneumoniae* subsp. *quasipneumoniae* 01A030, *K. quasipneumoniae* subsp. *similipneumoniae* 07A044, *K. variicola* GJ1 and the remaining strains of the bacterial panel described in the material and methods (Table S1).

The results pointed that, although KPC-142 strain was originally reported as *K. pneumoniae* before sequencing, it did not fit genomic species boundaries threshold set at 96%, and displayed ANI with the *K. pneumoniae* DSM 30104 type strain of 93.7% (Table S1). Rather, comparison to the type strains of *K. quasipneumoniae* subsp. *quasipneumoniae* (ANI of 96.52%) and *K. quasipneumoniae* subsp. *similipneumoniae* (ANI of 99.04%) revealed their relatedness (Table S1). These results are in line with those previously reported by Brisse et al. (2014), further supporting the classification of KPC-142 as subspecies *Klebsiella quasipneumoniae* subsp. *similipneumoniae* (hereafter referred as *Kqps*142). Misidentification of *Klebsiella* species by conventional clinical microbiology laboratory techniques was evidenced recently by Long et al. (2017), who demonstrated that almost 30% of tentatively identified *K. pneumoniae* strains in their study were in fact *K. quasipneumoniae*. Furthermore, through this ANI analysis, it can be inferred that the *K. pneumoniae* strains are slightly more similar to the *K. variicola* strains (ANI of 94.38%, median among the compared Kv strains) than to the *K. quasipneumoniae* (Kqq + Kqs) strains (ANI of 93.60%, median among the compared Kqq and Kqs strains) (Table S1). This observation correlates with the findings of Holt et al. (2015), where the *K. pneumoniae* and *K. variicola* phylogroups are closer to each other than *K. pneumoniae* and *K. quasipneumoniae* phylogroups (Holt et al., 2015).

**Figure 1:**
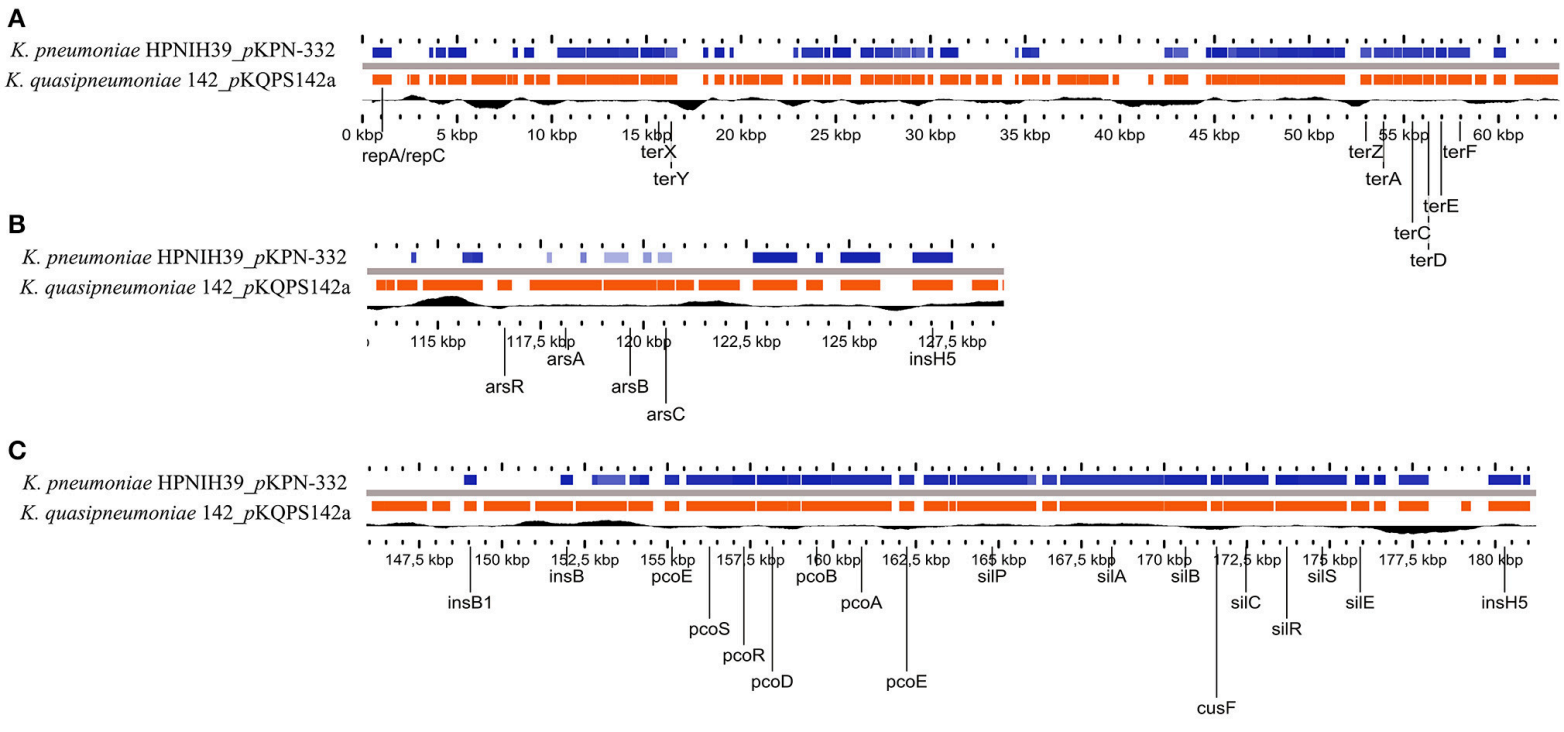
Genomic comparison between the plasmid *p*KQPS142a of *Kqps*142 and plasmid *p*KPN-332 of *K. pneumoniae* KPNIH39 performed by Gview program, using BlastN (>70% identity and *E*-values < 10^−5^). Black line represents GC content. **(A)** region of *ter* loci; **(B)** arsenical operon, and **(C)** copper-resistance operon and silver resistance genes.

Regarding the plasmid *p*KQPS142a with ~190 Kbp, searches using the PlasmidFinder database classified it as a conjugative IncFIB(K) plasmid, more similar to *p*KPN3 plasmid (50% coverage query, 98% maximum nucleotide identity) that has very narrow host range, limited to a predominantly human-associated sub-clade of *Klebsiella* (Kaplan et al., 2015). While *p*KPN3 plasmid is conjugative, *p*KQPS142a appears to have lost the genes for T4SS, and has an average of 50.71% G+C content, which is lower than the *K. quasipneumoniae* median of 57% (reported for 103 assemblies currently available in the NCBI/Genome database (www.ncbi.nlm.nih.gov/genome). This might be due to the considerable rearrangements with several mobile genetic elements throughout the plasmid sequence. Interestingly, *p*KQPS142a has multiple regions with high identity (70% query coverage, 98% maximum nucleotide identity) to plasmid *p*KPN-332 of *K. pneumoniae* KPNIH39, which was recovered from nosocomial infection during long-term patient colonization (Conlan et al., 2016) (Figure 1). Several key features are in common between *p*KQPS142a and *p*KPN-332, such as the RepFIB-like origin of replication (*repA/repC*), the *ter* loci (*terZABCDEF* and *terX*) that confer resistance to tellurium as well as resistance to bacteriophage and colicins (Taylor et al., 2002), a gene encoding for creatinase (creatine amidinohydrolase), an important medical enzyme that has been used for clinical diagnosis of renal function (Liu et al., 2015), the copper-resistance operon (*pcoABCDRS*) that encode an efflux system to both remove the toxic metal in excess and cytoplasmic copper management (Brown et al., 1995), fibrinolysin (plasminogen activator, *pla* gene) and the silver resistance genes (*sil*) (Figure 1). The predicted amino acid sequence of fibrinolysin (*pla* gene KPC142_05211) was highly similar (BLASTp >80%) to the plasminogen activator protease of *Yersinia pestis* CO92, which has a proteolytic activity responsible for the invasive character of plague and is considered an important virulence factor in this species (Lähteenmäki et al., 2001). In *Kqps*142 the genetic context of *pla* gene is characterized by two upstream transposases (KPC142_1834 and KPC142_1835) homologous to IS3 family of *Yersinia enterocolitica* (NC_008800), which could be involved in recombination events leading to spread of this *Yersinia* virulence factor among *Klebsiella* species.

The *sil* genes represent a major concern, since silver is commonly used in several types of medical devices, e.g., wound dressings, implants, catheter, and endotracheal tubes. While *Klebsiella* spp. are not primary wound pathogens, they have been linked with ventilator-associated pneumonia (Torres et al., 2009). In a recent Swedish tertiary hospital study with restricted consumption of silver-based products, the presence of *sil* operon genes was observed at high frequency, exclusively for members of the *Enterobacteriaceae* family and most common among *Enterobacter cloacae* and *K. oxytoca* isolates (Sütterlin et al., 2017). Furthermore, in the case of *K. pneumoniae* the silver resistance could be selected in a single step (i.e., by a single point mutation) from organisms harboring a repressed *sil* operon (Randall et al., 2015). Collectively these studies and the results showed here for a nosocomial isolate of *K. quasipneumoniae* subsp. *similipneumoniae* emphasize the advisement for the controlled use of silver-based products as well as the monitoring of silver resistance in hospital environments.

On the other hand, *p*KQPS142a could be considered as a multidrug plasmid, since it also carries the arsenical resistance operon (*ars*) that encode a specific detoxification pathway for arsenic extrusion (Achour-Rokbani et al., 2010) and a *sugE* gene encoding for quaternary ammonium compound efflux pump that at high-level expression leads to resistance to a subset of toxic quaternary ammonium compounds (Chung and Saier, 2002) (Figure 1). Also, we highlight another virulence factor encoded on *p*KQPS142a, i.e., the citrate-dependent iron(III) transport system FecABCDE, FecR, and FecI, where citrate is usually considered an external siderophore (Hussein et al., 1981).

**Figure 2:**
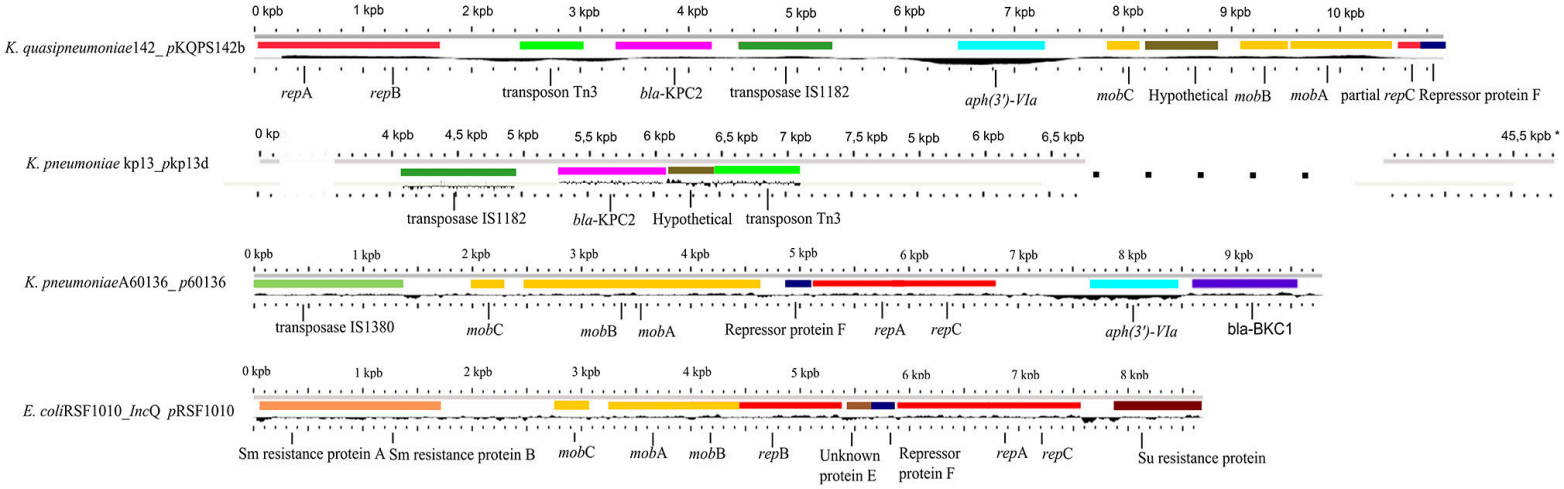
Comparison among the plasmids *p*KQPS142b of *Kqps*142, *K. pneumoniae* strain Kp13 (*p*Kp13d), *K. pneumoniae* strain A60136 (*p*60136), and *E. coli* (IncQ *p*RSF1010). Homologous genes to plasmid *p*KPC142b carrying *bla*_KPC−2_ were obtained by BLASTn, and represented by the same color in other species, each color defined to a specific gene. Black line represents the GC content. Features of each plasmid were obtained using the GView program, with modifications.^*^The *p*Kp13d is significantly larger than other compared elements. The genome length scales were adapted to emphasize only the region with similarity to *p*KQPS142b.

Of special interest in *Kqps*142 is the plasmid *p*KQPS142b carrying *bla*_KPC−2_. We identified that this small plasmid has homology with the broad host-range IncQ1 plasmid pRSF1010 of *E. coli* (Scholz et al., 1989) in the regions involved in plasmid replication and mobilization, i.e., *mob* and *rep* genes (Figure 2). Also, *p*KQPS142b shows an extensive region of high identity (99% identity, from *mobA, mobB*, repression protein F, *repA, repB* to *aph*(3')-VIa) with the IncQ1-like *p*60136 plasmid of *K. pneumoniae* strain A60136. The *p*60136 carries a novel β-lactamase, namely Brazilian *Klebsiella* carbapenemase-1 (BKC-1) and was isolated in São Paulo, Brazil in 2009 (Nicoletti et al., 2015). While *p*60136 carries BKC-1, *p*KPC142b harbors *bla*_KPC−2_ (KPC142_06049), flanked by an upstream IS*Kpn*6 transposase from IS1182 family and a downstream transposon resolvase Tn3. This plasmid region has around 3 Kbp and showed high similarity to the homologous region of *p*Kp13d of *K. pneumoniae* Kp13 isolated in Southern Brazil in 2009, whose complete genome has been previously reported by our group (Ramos et al., 2014) (Figure 2).

Nicoletti et al. demonstrated that *p*60136 is a non-transferable plasmid by conjugation from *E.coli* transformant cells, but it can be mobilized at a high frequency by helper conjugative plasmids (Nicoletti et al., 2015). However, we found no evidence of T4SS genes in the sequence of the largest plasmid *p*KQPS142a that would support plasmid-mediated conjugation functions provided in *trans*.

On the other hand, we found an integrative conjugative element ICE*Pm1* that is conserved in *Proteus mirabilis, Providencia stuartii*, and *Morganella morganii* (Flannery et al., 2009) and it was exclusive when compared to a panel of 35 strains representative of each *Klebsiella* phylogroup (Table 1 and see R7 in Figure 4). In *Kqps*142, the ICE*Pm1* is a 92,220 bp region that extends from a phage integrase encoding gene (KPC142_03753) to a chromosome partitioning-related encoding gene (KPC142_05483), which contains a core segment showing homology to a T4SS for DNA transfer (KPC142_03739 to KPC142_03716). Interestingly, and in a fashion similar to plasmids, ICEs have the ability to transfer other mobile elements in *trans* (Meyer, 2009). Another ICE, namely R391/SXT family of ICEs of *Vibrio cholerae* O139 has been demonstrated to mobilize the IncQ plasmid RSF1010 in an *oriT*-independent manner. This and further studies suggested that other mechanisms must account for this unexpected *oriT*-independent mobilization of RSF1010, which are still under investigation (Hochhut et al., 2000; Poulin-Laprade et al., 2015). Also, ICE*Pm1* and STX are compatible, since both ICEs were found in a single bacterium of *P. mirabilis* HI4320 (Flannery et al., 2011). These findings raise the question whether the ICE*Pm1* is capable of transferring the *p*KQPS142b (IncQ plasmid) in *trans*, further aiding in the dissemination of *bla*_KPC−2_.

Additionally, in *p*KQPS142b the inverted right repeat (IRR) of transposon Tn3 was identified between IS*Kpn*6 and *bla*_KPC−2_, but as previously reported in *p*Kp13d of *K. pneumoniae* Kp13 no inverted left repeat (IRL) was detected. This can be regarded as evidence of horizontal spread of *bla*_KPC−2_ gene between *K. pneumoniae* and *K. quasipneumoniae* by recombination events involving IS*Kpn*6 and the flanking Tn3-family sequences and facilitated by the broad host-range mobilizable IncQ1 plasmid. In addition, the *bla*_KPC−2_-carrying IncQ *p*KQPS142b plasmid found here is highly relevant in view of few reports of plasmids associated to the spread of such relevant resistance gene (Carattoli, 2011; Nicoletti et al., 2015; Mollenkopf et al., 2017).

### Analysis of multilocus sequence and capsule typing

Multilocus sequence typing (MLST) analysis revealed a novel combination of known *K. pneumoniae* MLST alleles (*gapA*−18, *infB*−22, *mdh*−56, *pgi*−61, *phoE*−74, *rpoB*−38, *tonB*−99), with ST2736 being the closest sequence type, differing only in *pgi* (allele 16) and *phoE* (allele 11) loci, with ST1032, ST1361, ST1413, ST1703, ST2119, and ST2137 differing in three alleles.

**Figure 3:**
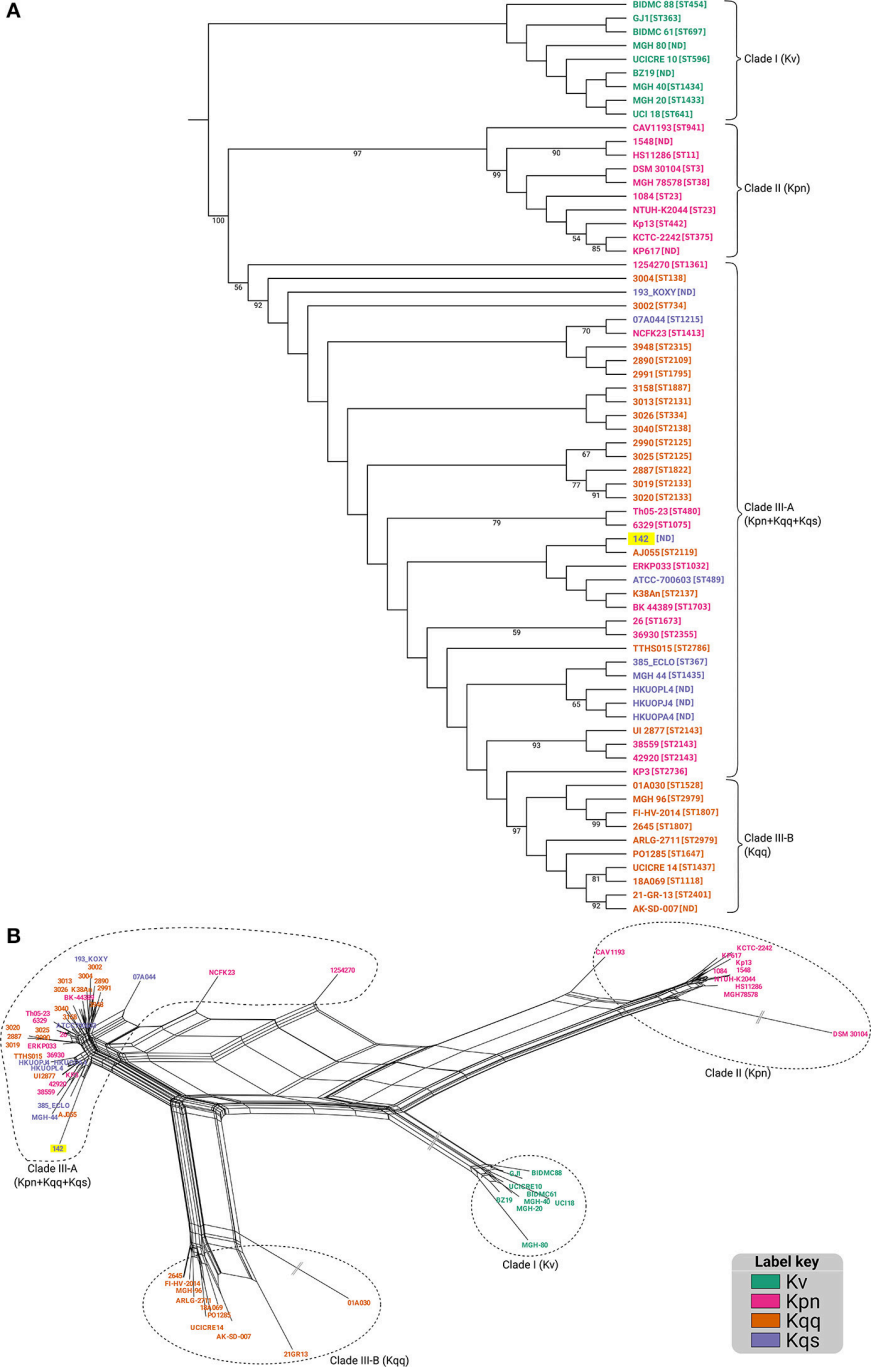
**(A)** Maximum likelihood tree for the matrix consisting of the concatenated allele sequences for seven housekeeping genes (*gapA, infB, mdh, pgi, phoE, rpoB*, and *tonB*) loci, constructed with PhyML, using the GTR+I+G model of nucleotide substitution. 1,000 bootstrap replicates were calculated to assess robustness. 1,000 bootstrap replicates were calculated to assess robustness, and support values >50% are shown. **(B)** Split decomposition analysis using the seven MLST alleles reveals a network-like structure, suggestive of recombination events. “||” represent long branches that were truncated to fit (see Figure S3 for the complete network). In both panels, KPC-142 is labeled on a yellow background and in the (panel **A)** the ST number of each isolate/strain is in brackets.

Maximum likelihood phylogenetic analysis performed using the concatenated MLST genes showed the existence of three main groups. The first, Clade I, comprising all *K. variicola* sequences; Clade II grouped only *K. pneumoniae* strains, while Clade III was composed by strains from both *K. quasipneumoniae* subspecies, as well as some *K. pneumoniae* isolates (Figure 3A). The phylogenetic placement of *Kqps*142 was closer to Clade III strains, which included all *K. quasipneumoniae* strains, reinforcing its classification as *K. quasipneumoniae. Kqps*142 grouped with *K. quasipneumoniae* subsp. *quasipneumoniae* AJ055, an ST2119 isolate differing by three MLST alleles. Also in Clade III were strains with STs differing by few alleles compared to *Kqps*142, such as *K. pneumoniae* ERKP033 (ST1032), *K. pneumoniae* BK44389 (ST1703), and *K. quasipneumoniae* subsp. *quasipneumoniae* K38An (ST2137). In order to better elucidate the complex relationships between these strains, a split decomposition analysis was performed, which yielded a network-like structure suggestive of high levels of recombination in the studied population (Figure 3B and Figure S3). The pairwise homoplasy index (phi) was significant (*p*-value = 0.0), indicating strong statistical support for the recombination events in the split network. In this network, the three main clades identified in the maximum likelihood tree were recapitulated, however, the relationships within Clade III could be better delineated, as this branch was separated into Clade III-A (grouping both subspecies of *K. quasipneumoniae* and some *K. pneumoniae* strains) and Clade III-B (containing only *K. quasipneumoniae* subsp. *quasipneumoniae* strains) (Figure 3B). These latter strains could be regarded as closer to the type strain of this subspecies (strain 01A030), while those in Clade III-A, including *Kqps*142, probably underwent more recent recombination events leading to their clustering in the split network.

*In silico* capsule typing was performed using a standard nomenclature to refer to the capsular locus type (K-locus are designated as KL) (Wyres et al., 2016) and comparisons against the *K. pneumoniae* BIGSdb database (http://bigsdb.web.pasteur.fr). This identified that *Kqps*142 belongs to KL16 type, containing 22 genes with high similarity to the best match reference locus (97.2% identity and 100% coverage) with *manB, manC*, and *wzc* allele type 17. The capsular region also carries a novel *wzi*, a variant that differ to the most closely related allele 60 in two bases: ^444^T^444^G (base depth of coverage 63 without variants) ^447^C^447^T (base depth of coverage 63 without variants), raising the possibility that if this *wzi* allele is indeed novel, it could associate to the K16 capsular locus.

### Antimicrobial susceptibility tests associated with resistance factors

The antimicrobial susceptibilities of *Kqps*142 are summarized in Table 2. The isolate presented resistance to all tested beta-lactam antibiotics (ampicillin, ampicillin-sulbactam, cefepime, cefoxitin, ceftazidime, ceftriaxone, cefuroxime, ertapenem, imipenem, meropenem, and piperacilin-tazobactam). Susceptibilities were identified for amikacin (aminoglycoside), gentamicin (aminoglycoside), ciprofloxacin (fluoroquinolone), tigecycline, and colistin (polymyxin E). Correlation between phenotypic antibiotic susceptibility and the resistome, particularly for beta-lactam resistance, showed that besides KPC-2 (gene locus KPC142_06049, found in *p*KQPS142b), *Kqps*142 also harbors the chromosomal gene encoding for OKP-B-6 (KPC142_02249). While *p*KQPS142b carries *aph*(3')-Via (synonymous to *aphA6*), the MICs of the aminoglycoside antibiotics tested in this strain were low (amikacin, MIC 8 mg/L and gentamicin, MIC ≤ 1 mg/L). Similarly, Yoon et al. also observed an unexpected amikacin susceptibility of the *aphA6*-carrying *Acinetobacter guillouiae* strains, and the authors correlated this phenotype with low level of gene expression (Yoon et al., 2014), which remains to be tested in the isolate KPC-142.

**Table 2:** Antimicrobial susceptibility profile of KPC-*Kqps*142.

**Antibiotic**	***Kqps142* MIC (mg/L)**
Amikacin^b^	8 (S)^a^
Ampicillin^c^	≥32 (R)
Ampicillin-sulbactam^c^	≥32 (R)
Cefepime^c^	16 (R)
Cefoxitin^c^	≥64 (R)
Ceftazidime^c^	16 (R)
Ceftriaxone^c^	32 (R)
Cefuroxime^c^	≥64 (R)
Ciprofloxacin^d^	≤ 0.25 (S)
Colistin^e^	≤ 0.5 (S)
Ertapenem^c^	≥8 (R)
Gentamicin^b^	≤ 1 (S)
Imipenem^c^	≥16 (R)
Meropenem^c^	≥16 (R)
Piperacilin-tazobactam^c^	≥128 (R)
Tigecycline	≤ 0.5 (S)

a Interpretation is in parenthesis;

b Aminoglycoside;

c Beta-lactam;

d Fluoroquinolone;

e *polymyxin E*.

### Chromosomal gene content comparisons among the analyzed *Klebsiella* strains

We compared the chromosomal gene content between *Kqps*142 and strains belonging to *K. quasipneumoniae* subsp. *similipneumoniae* (phylogroup II-B), *K. quasipneumoniae* subsp. *quasipneumoniae* (phylogroup II-A), *K. pneumoniae* (phylogroup I), and *K. variicola* (phylogroup III), supporting a panel of 36 strains representative of each *Klebsiella* phylogroup (Holt et al., 2015).

Proteinortho co-orthologous clustering analysis revealed a total of 1,049 core genes conserved in all 36 genomes (Table S2), whereas we found 1,597 genes that were shared by ≥ 97% of the genomes that composed our bacterial panel of strains belonging to phylogroups KpI, KpII, and KpIII. We identified a pangenome of 18,330 unique protein-coding sequences among the 36 analyzed *Klebsiella* genomes. These results are commensurable with those shown by Holt et al. (2015), who identified a pangenome of 29,886 unique protein-coding sequences among 328 *K. pneumoniae genomes* and revealed an open pangenome (Holt et al., 2015). Concerning the “exclusive” genes, a total of 9,795 genes had no orthologos among the 36 genomes (yielding a median of 272 “exclusive” genes per genome). Particularly, this comparative analysis revealed 181 “exclusive” genes in the genome of isolate *Kqps*142, most of which are genes of unknown function, or genes related to ICE*Pm1* and capsule biogenesis (*wzb, wzc, wzx*, and *wzy*) (Table S2).

**Figure 4:**
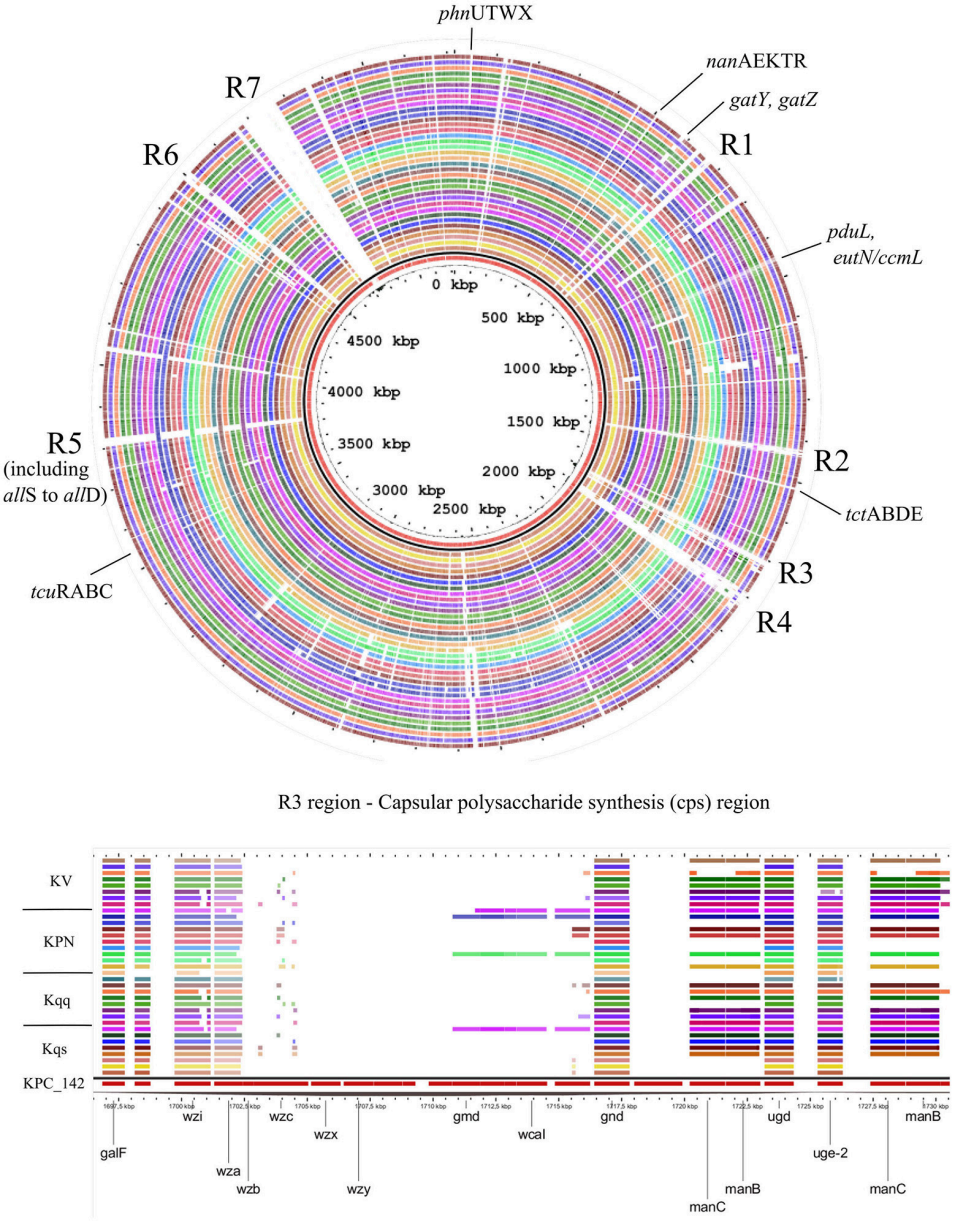
Genomic Atlas showing the common or exclusives chromosomal genes of *Kqps*142 (inner circle, in red), in comparison to: eight *K. quasipneumoniae* subsp*. similipneumoniae* strains (in order, inner to outer circles—HKUOPA4, HKUOPJ4, HKUOPL4, ATCC 700603, 07A044, MGH44, 193 KOXY, and 385 ECLO); nine *K. quasipneumoniae* subsp*. quasipneumoniae* (in order, inner to outer circles—UCICRE14, 01A030, FI_HV_2014, MGH96, 18A069, ARLG2711, PO1285, AKSD007, and 21_GR_13); nine *K. pneumoniae* (in order, inner to outer circles—DSM_30104, KP13, 1084, CAV1193, HS11286, KCTC-2242, KP617, MGH 78578, and NTUH-K2044); and nine *K. variicola* (in order, inner to outer circles–BZ19, GJ1, UCICRE10, MGH40, MGH20, UCI18, BIDMC61, BIDMC88, and MGH80). Comparison was obtained by Gview program, using BlastN (>70% identity and *E*-values < 10^−5^). Black line represents GC content. Regions containing interesting genes or features (named R1 to R7) are highlighted. R7 contains phage sequences and the integrative conjugative element ICE*Pm1*. Genes of capsular polysaccharide synthesis are shown in details. Kqs, *K. quasipneumoniae* subsp*. similipneumoniae*; Kqq, *K. quasipneumoniae* subsp. *quasipneumoniae;* Kpn, *K. pneumoniae;* and Kv, *K. variicola*.

Major chromosomal features, common or exclusive, among compared *Klebsiella* strains are depicted in Figure 4. Genome Atlas shows that *Kqps*142 harbors seven main regions of genomic plasticity in comparison to other 35 *Klebsiella* genomes, almost all of which with variations in G+C content (except R5). R3 presents the capsular polysaccharide biosynthesis genes, while R1, R2, R4, and R6 are composed by ORFs involved in phage integration. R5 includes the allantoin operon and R7 contains phage sequences and the integrative conjugative element ICE*Pm1*.

**Figure 5:**
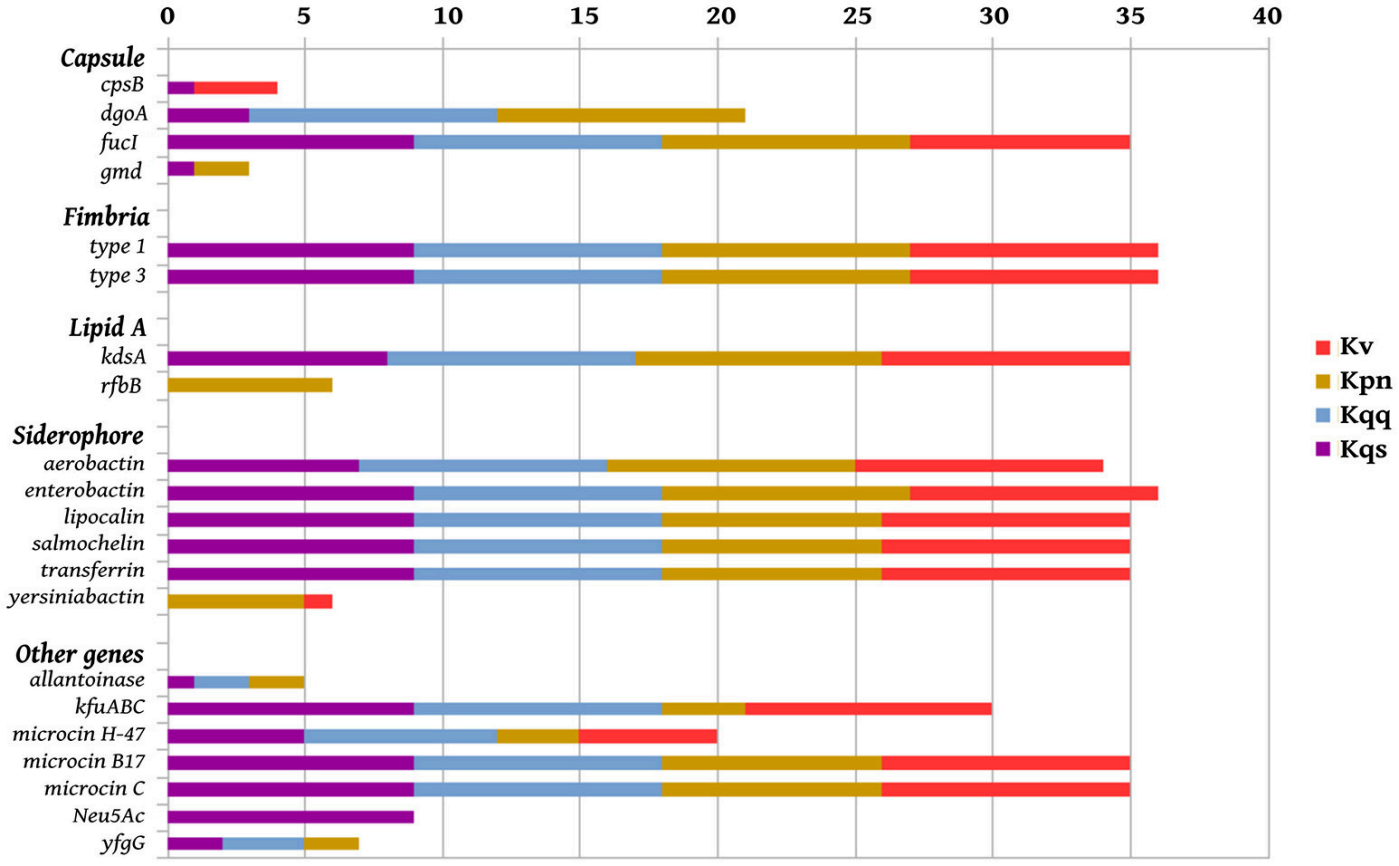
Virulence genes in *Klebsiella* strain panel, including *Kqps*142. Presence of the virulence genes per genome (from 0 to 9) of *K. pneumoniae* (*Kpn*), *K. quasipneumoniae* subsp. *quasipneumoniae (Kqq), K. quasipneumoniae* subsp. *similipneumoniae (Kqs), and K. variicola (Kv)*.

Virulence genes identified in the 36 genomes from each *Klebsiella* phylogroup, including the isolate *Kqps*142, are shown in Figure 5. These included genes associated with the biosynthesis of siderophores, fimbriae, lipid A, capsule, or genes encoding for microcin, allantoinase, and the ferric uptake operon *kfuABC*, which have been reported as virulence factors of *K. pneumoniae* on the basis of murine models of infection (Paczosa and Mecsas, 2016). Most of these genes were detected in all *Klebsiella* phylogroups, since the majority of compared *Klebsiella* strains were isolated from human infections (Table 1). However, a few genes were identified in just one or two *Klebsiella* phylogroups, namely those genes encoding for the siderophore yersiniabactin (found in five *K. pneumoniae* and one *K. variicola*), capsular *gmd* gene (found in only one *K. pneumoniae* and three *K. quasipneumoniae* subsp. *quasipneumoniae*), *rfbB* gene (found only in six *K. pneumoniae*), and the N-acetyl-neuraminic acid gene cluster (found exclusively in all *K. quasipneumoniae* subsp. *similipneumoniae*) (see also Table 3).

**Table 3:** Genomic characteristics of the *K. quasipneumoniae* subsp. *similipneumoniae* (Kqs), *K. quasipneumoniae* subsp. *quasipneumoniae* (Kqq), *K. pneumoniae* (Kpn), and *K. variicola* (Kv) strains, related to metabolism of carbon, nitrogen, and phosphorous sources and other genes.

***Klebsiella* species**	**Strain**	**Neu5Ac catabolism pathway^a^**	**D-tagatose catabolism pathway^b^**	**Tricarballylate catabolism pathway^c^**	**D-Psicose catabolism pathway^d^**	**2-AEP transport and utilization *(phnUTWX)***	**Allantoin catabolism pathway^e^**	**TTT^f^**
Kps	*Kqps*142	Yes	Yes	Yes	–	Yes	Yes	Yes
	07A044	Yes	Yes	Yes	–	Yes	–	Yes
	ATCC700603	Yes	Yes	Yes	–	Yes	–	Yes
	HKUOPA4	Yes	–	Yes	–	Yes	–	Yes
	HKUOPJ4	Yes	–	Yes	–	Yes	–	Yes
	HKUOPL4	Yes	–	Yes	–	Yes	–	Yes
	MGH44	Yes	–	Yes	–	Yes	–	Yes
	193_KOXY	Yes	Yes	Yes	–	Yes	–	Yes
	385_ECLO	Yes	–	Yes	–	Yes	–	Yes
Kqq	01A030	–	–	Yes	–	Yes	–	Yes
	UCICRE14	–	–	Yes	Yes	Yes		Yes
	FIHV2014	–	–	Yes	Yes	Yes	Yes	Yes
	MGH96	–	–	Yes	–	Yes	–	Yes
	18A069	–	–	Yes	–	Yes	–	Yes
	ARLG2711	–	–	Yes	–	Yes	–	Yes
	PO1285	–	–	Yes	–	Yes	Yes	Yes
	AK_SD_007	–	–	Yes	–	Yes	–	Yes
	21GR13	–	–	Yes	–	Yes	–	Yes
Kpn	DSM30104	–	–	–	–	Yes	–	–
	KP13	–	–	–	Yes	Yes	–	–
	1084	–	Yes	–	Yes	Yes	Yes	–
	CAV1193	–	Yes	–	Yes	Yes	–	–
	HS11286	–	Yes	–	Yes	Yes	–	–
	KCTC2242	–	–	–	–	Yes	–	–
	KP617	–	–	–	–	Yes	–	–
	MGH78578	–	Yes	–	Yes	Yes	–	–
	NTUHK2044	–	Yes	–	Yes	Yes	Yes	–
Kv	GJ1	–	–	Yes	–	–	–	–
	BZ19	–	Yes	Yes	–	–	–	–
	UCICRE10	–	Yes	Yes	–	–	–	–
	MGH40	–	–	Yes	–	–	–	–
	MGH20	–	–	Yes	–	–	–	–
	UCI18	–	Yes	Yes	–	–	–	–
	BIDMC61	–	–	Yes	–	–	–	–
	BIDMC88	–	Yes	Yes	–	–	–	–
	MGH80	–	–	Yes	–	–	–	–

a Ne5Ac catabolism pathway consisting of nanA, nanE, nanK, nanT, and nanR genes.

b D-tagatose catabolism pathway consisting of both gatY and gatZ genes;

c Based on the presence of tcuRABC genes.

d Based on the presence of eight genes for D-psicose utilization (partial in FIHV2014) (Blin et al., 2017).

e Based on the presence of 13 genes from allS to allD (Chou et al., 2004).

f *Based on the presence of tctA, tctB, tctD, tctE genes. Ne5Ac, N-acetyl-neuraminic acid; 2-AEP, 2-aminoethylphosphonate; TTT, tripartite tricarboxylate transporter*.

Table 3 shows the gene content related to the most relevant metabolic pathways among 36 strains representative of each *Klebsiella* phylogroup, including the isolate *Kqps*142. Regarding the protein-encoding genes shared only by *K. quasipneumoniae* subsp. *similipneumoniae* strains are of special interest the N-acetyl-neuraminic acid gene cluster encompassing *nanTEAR* (KPC142_01465 to KPC142_01468) and *nanK* (KPC142_01464), which are shared by the nine analyzed strains of *K. quasipneumoniae* subsp. *similipneumoniae* (Table 3, Figure 4 and Table S2). Blin et al. (2017) showed that the ability to use N-acetyl-neuraminic acid (Neu5Ac) could be a phenotypic marker for *K. quasipneumoniae* subsp. *similipneumoniae* (Blin et al., 2017). Neu5Ac is especially abundant in the epithelial mucus of eukaryotic host and bacteria can scavenge this canonical sialic acid, for either catabolism and/or sialylation of their cell surface, which serves as a key determinant of pathogenesis (Haines-Menges et al., 2015) (see also Figure S4).

The metabolism of allantoin is used by bacteria as a source of carbon and nitrogen and is considered an important determinant of virulence in *K. pneumoniae* (Paczosa and Mecsas, 2016), since the operon genes from *allS* to *allD* are upregulated in hypervirulent (HV) *K. pneumoniae* strains compared to classical strains (Liu et al., 1986; Chou et al., 2004). The presence of allantoin operon genes among the 36 analyzed *Klebsiella* strains was very rare, limited only to *Kqps*142, *K. quasipneumoniae* subsp. *quasipneumoniae* strains FIHV2014 and PO1285 and *K. pneumoniae* strains 1084 and NTUH K2044 (Table 3 and Figure 4). Indeed, strain NTUH K2044 causes liver abscess and meningitis (Wu et al., 2009), strain FIHV2014 was described as a hypermucoviscosity-positive clinical isolate (Arena et al., 2015) and although 1084 was described as a hypermucoviscosity-negative clinical isolate it was capable of inducing liver abscesses in a murine model (Lin et al., 2011, 2012). On the other hand, while strains PO1285 (NCBI BioProject PRJNA351846) and *Kqps*142 are clinical isolates from human infections, their virulence *in vivo* remains to be investigated.

The genes involved in the metabolism of D-tagatose were not found among nine *K. quasipneumoniae* subsp. *quasipneumoniae* genomes and were rare among Kqq, Kqs, and Kpn strains (Table 3 and Figure 4). These genes encode for tagatose-bisphosphate aldolase subunit GatY (EC number 4.1.2.40) (KPC142_01383) and the subunit GatZ (KPC142_01387) (Table S2) that functions as a chaperone-like for the proper and stable folding of GatY (Brinkkötter et al., 2002). Fermentation and substrate assimilation tests were previously performed showing that *K. quasipneumoniae* subsp. *similipneumoniae* strains produce acid from D-tagatose and can grow on this sugar (Brisse et al., 2014; Blin et al., 2017).

The *tcuRABC* genes that encode functions needed for the utilization of tricarballylate as a carbon and energy source were absent only in the nine analyzed *K. pneumoniae* strains (Table 3 and Figure 4). These genes were described in the *Salmonella enterica* serovar Typhimurium LT2 (Lewis et al., 2004) and the utilization of tricarballylic acid was experimentally demonstrated as very rare in *K. pneumoniae* (Blin et al., 2017). A putative operon involved in the ability to use D-psicose was more frequent in the *K. pneumoniae* strains and it was also identified in two *K. quasipneumoniae* subp. *quasipneumoniae* genomes (UCICRE14 and FIHV2014, Table 3), which are in agreement with reported phenotypic findings (Blin et al., 2017).

An operon (*phnUTWX*) involved in the metabolism of 2-aminoethylphosphonate (2-AEP) as phosphorus source was only absent in the nine analyzed *K. variicola* strains (Table 3 and Figure 4). In *Enterobacter aerogenes* and *Salmonella enterica* subsp. *enterica* serovar Typhimurium, 2-AEP catabolism might be controlled as part of the Pho regulon and these bacteria can use 2-AEP as an alternative source of phosphate only under conditions of P limitation (Jiang et al., 1995), and in *Pseudomonas putida* str. NG2 and *Pseudomonas aeruginosa* str. PAO1 2-AEP can be mineralized as the sole C, N and P source (Ternan and Quinn, 1998; McGrath et al., 2013).

In bacteria, the tripartite tricarboxylate transporter (TTT) TctCAB is involved in the citrate uptake, which then can be utilized as a carbon and energy source by bacterial cell. The system was well-studied in *Bordetella pertussis* (Antoine et al., 2003), *Salmonella typhimurium* (Widenhorn et al., 1989), and *Corynebacterium glutamicum*, a nonpathogenic species that is of interest due to its biotechnological importance as a producer of L-glutamate and L-lysine (Brocker et al., 2009). Here, we found the *tctCAB* operon genes were restricted to strains belonging to *K. quasipneumoniae* species (Table 3 and Figure 4).

Also, protein-encoding genes involved in two pathways, i.e., 1,2- propanediol (1,2-PD) and ethanolamine (Eut) utilization were found in almost all compared strains (except for *K. pneumoniae* DSM 30104, Table S2 and Figure 4). Both the coenzyme B12-dependent catabolism of 1,2-PD (PduL EC: 2.3.1.222, KPC142_03505) and B12-dependent degradation of ethanolamine (*eut* operon) use the bacterial microcompartment (BMC) structures encoded by *pduA* (KPC142_03506) and *eutN*/*ccmL* (KPC142_03510), respectively. The BMC function is to optimize biochemical pathways by confining toxic by-products or volatile metabolic intermediates (Bobik et al., 2015). The ethanolamine and 1,2-PD degradations seem to be important to enteric pathogenesis (Thiennimitr et al., 2011) and their relevance in the pathogenesis or fitness of *Klebsiella* species still remains to be experimentally examined.

## Conclusions

While the association of *K. pneumoniae* as responsible for infections in nosocomial settings is well-established, there are currently few reports associating *K. quasipneumoniae* as culprit in this context. Here, we reported the complete, closed genome of isolate *K. quasipneumoniae* subsp. *similipneumoniae* KPC-142, composed of one chromosome and two plasmids. To the best of our knowledge, this is the first complete genome of a clinical isolate of this subspecies harboring both beta-lactamases KPC-2 and OKP-B-6 responsible for a nosocomial infection from South America. The identification of multiple virulence and resistance factors coded in this genome, along with its drug resistance spectrum, reveals that, as in *K. pneumoniae*, this bacterium has great potential to establish infection and overcome the action of antibiotics currently used to treat these patients. The genomic characterization of *K. quasipneumoniae* strains will contribute to improve diagnostics and to the understanding of the epidemiological relevance of the dissemination of this *Klebsiella* species.

## Author contributions

CMA, DC, and RC provided the isolate *Kqps*142 and performed initial bacterial identification and susceptibility tests. Randomly Amplified Polymorphic DNA (RAPD) analysis was performed by RC and DC. ACV conceived the sequencing strategy. Complete genome assembly was carried out by LA and GL. Manual annotation and bioinformatic analyses were performed by ATR, FMC, LC, LA, MN, PPR, and RS. The manuscript was prepared by MN, PPR, FMC, and ATR. All authors read and approved the final manuscript.

### Conflict of interest statement

The authors declare that the research was conducted in the absence of any commercial or financial relationships that could be construed as a potential conflict of interest.

## Supplementary material

Table S1 Average nucleotide identity (ANI) comparisons of *Klebsiella* strain panel, including *Kqps*142. ANI was calculated using BLASTn (ANIb) in JSpeciesWS, using *K. quasipneumoniae* subsp. *quasipneumoniae* 01A030, *K. quasipneumoniae* subsp. *similipneumoniae* 07A044, *K. pneumoniae* subsp. *pneumoniae* DSM 30104, as reference type strains, in addition to strain *K. variicola* GJ1. An ANI threshold of ≥96% was considered to delineate species boundaries.

Table S2 (A) Total clusters of co-orthologous coding sequences generated from comparisons among 36 *Klebsiella* strains. **(B)** Clusters of co-orthologous coding sequences of core genes conserved in all 36 genomes. **(C)** List of “exclusive” genes in the genome of the isolate *Kqps*142. Comparisons were performed through Proteinortho co-orthologous clustering using BLASTp with an identity/coverage cutoff of >90% and E-values < 10.5.

Figure S1 RAPD analysis. Initially, 83 KPC isolates carrying the blaKPC gene were collected from nosocomial infections in Minas Gerais state (Southeast Brazil) in 2011. These isolates were preliminarily clustered by Randomly Amplified Polymorphic DNA (RAPD) with primer M13 (5′-GTAAAACGACGGCCAG-3′) using previously published reaction conditions (Wong et al., 1994). Seven clusters were disclosed and the isolate named 142, which displayed the most divergent DNA pattern by RAPD cluster analysis selected for sequencing analysis (Figure S1). Fragment sizes in RAPD fingerprints were estimated using GelAnalyzer (GelAnalyzer.com) and a distance matrix was calculated with MST (Coimbra et al., 2010). Distance tree was built with NEIGHBOR program within the PHYLIP package (Felsenstein, 1989) and drawn with MEGA 6.0 (Tamura et al., 2013). A red dot highlights the isolate KPC-142 selected for whole-genome sequencing.

Figure S2 An illustration of the assembly and gap-closing strategies. Gaps intra- and inter-scaffolds were resolved using an hybrid assembly strategy with the SPAdes and Newbler programs, with local assemblies in gap regions using Newbler.

Figure S3 Split decomposition analysis using the seven MLST alleles reveals a network-like structure, suggestive of recombination events.

Figure S4 Schematic representation of the utilization of N-acetyl-neuraminic acid (Neu5Ac) in a hypothetical *K. quasipneumoniae subsp. similipneumoniae* cell. The Kqs cell has potentially the ability to scavenge host Neu5Ac and use it for catabolism and/or sialylation of its cell surface structures, since it carries the gene encoding for a sialidase (*nanH*), a porin (*nanT*), sialic acid catabolism (SAC), and cell surface sialylation (Haines-Menges et al., 2015). The genome of Kqq, Kpn, and Kv strains carries *nanH* encoding for a sialidase and genes encoding for cell surface sialylation, but the genes encoding for SAC are absent, so these strains can potentially use Neu5Ac only for sialylation of their cell surface structures. Kqs, *K. quasipneumoniae* subsp*. similipneumoniae*; Kqq, *K. quasipneumoniae* subsp*. quasipneumoniae;* Kpn, *K. pneumonia;* and Kv, *K. variicola*
