# Supplementary material for: Comparative Genomic Analysis of a Clinical Isolate of Klebsiella quasipneumoniae subsp. similipneumoniae, a KPC-2 and OKP-B-6 Beta-Lactamases Producer Harboring Two Drug-Resistance Plasmids from Southeast Brazil
Source: Front Microbiol. 2018 Feb 16;9:220. doi: 10.3389/fmicb.2018.00220 (PMC5820359; doi:10.3389/fmicb.2018.00220)
Supplement: Figure S1 — RAPD analysis. Initially, 83 KPC isolates carrying the blaKPC gene were collected from nosocomial infections in Minas Gerais state (Southeast Brazil) in 2011. These isolates were preliminarily clustered by Randomly Amplified Polymorphic DNA (RAPD) with primer M13 (5′-GTAAAACGACGGCCAG-3′) using previously published reaction conditions (Wong et al., 1994). Seven clusters were disclosed and the isolate named 142, which displayed the most divergent DNA pattern by RAPD cluster analysis selected for sequencing analysis (Figure S1). Fragment sizes in RAPD fingerprints were estimated using GelAnalyzer (GelAnalyzer.com) and a distance matrix was calculated with MST (Coimbra et al., 2010). Distance tree was built with NEIGHBOR program within the PHYLIP package (Felsenstein, 1989) and drawn with MEGA 6.0 (Tamura et al., 2013). A red dot highlights the isolate KPC-142 selected for whole-genome sequencing. [file Image1.pdf]

Supplementary Material

Comparative genomic analysis of a clinical isolate of *Klebsiella quasipneumoniae* subsp. *similipneumoniae*, a KPC-2 and OKP-B-6 beta-lactamases producer harboring two drug-resistance plasmids from Southeast Brazil

Authors: Marisa F. Nicolás[1†], Pablo Ivan Pereira Ramos[2†], Fabíola Marques de Carvalho[1†], Dhian Renato Almeida Camargo[3], Carlene de Fátima Morais Alves[3], Guilherme Loss de Morais[1], Luiz G. P. Almeida[1], Rangel C. Souza[1], Luciane Prioli Ciapina[1], Ana Carolina Vicente[4], Roney S. Coimbra[5], Ana Tereza Ribeiro de Vasconcelos[1*]

*** Correspondence:** Corresponding Author: E-mail: atrv@lncc.br

Figure S1. RAPD analysis. Initially, 83 KPC isolates carrying the bla_{KPC} gene were collected from nosocomial infections in Minas Gerais state (Southeast Brazil) in 2011. These isolates were preliminarily clustered by Randomly Amplified Polymorphic DNA (RAPD) with primer M13 (5'-GTAAAACGACGGCCAG-3') using previously published reaction conditions (Wong et al., 1994). Seven clusters were disclosed and the isolate named 142, which displayed the most divergent DNA pattern by RAPD cluster analysis selected for sequencing analysis (Figure S1). Fragment sizes in RAPD fingerprints were estimated using GelAnalyzer (GelAnalyzer.com) and a distance matrix was calculated with MST (Coimbra et al., 2010). Distance tree was built with NEIGHBOR program within the PHYLIP package (Felsenstein, 1989) and drawn with MEGA 6.0 (Tamura et al., 2013). A red dot highlights the isolate KPC-142 selected for whole-genome sequencing.

frontiers

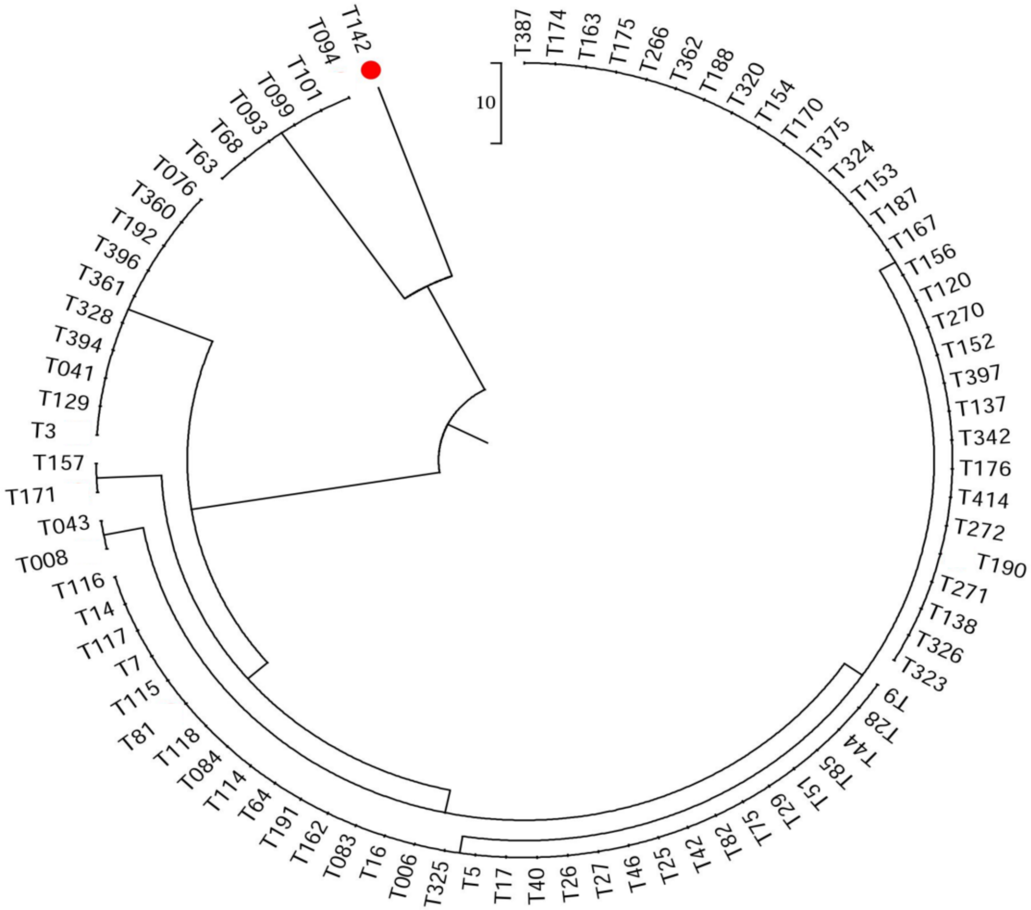
10
T142
T094
T101
T099
T093
T68
T63
T076
T360
T192
T396
T361
T328
T394
T041
T129
T3
T157
T171
T043
T008
T116
T14
T117
T7
T115
T81
T118
T084
T114
T64
T191
T162
T083
T16
T006
T325
T5
T17
T40
T26
T27
T46
T25
T42
T82
T75
T29
T51
T85
T44
T28
T9
T323
T326
T138
T271
T190
T272
T414
T176
T342
T137
T397
T152
T270
T120
T156
T167
T187
T153
T324
T375
T170
T154
T320
T188
T362
T266
T175
T163
T174
T387
