# Supplementary material for: Comparative Genomic Analysis of a Clinical Isolate of Klebsiella quasipneumoniae subsp. similipneumoniae, a KPC-2 and OKP-B-6 Beta-Lactamases Producer Harboring Two Drug-Resistance Plasmids from Southeast Brazil
Source: Front Microbiol. 2018 Feb 16;9:220. doi: 10.3389/fmicb.2018.00220 (PMC5820359; doi:10.3389/fmicb.2018.00220)

Supplementary Material

Comparative genomic analysis of a clinical isolate of *Klebsiella quasipneumoniae* subsp. *similipneumoniae*, a KPC-2 and OKP-B-6 beta-lactamases producer harboring two drug-resistance plasmids from Southeast Brazil

Authors: Marisa F. Nicolás[1†], Pablo Ivan Pereira Ramos[2†], Fabíola Marques de Carvalho[1†], Dhian Renato Almeida Camargo[3], Carlene de Fátima Morais Alves[3], Guilherme Loss de Morais[1], Luiz G. P. Almeida[1], Rangel C. Souza[1], Luciane Prioli Ciapina[1], Ana Carolina Vicente[4], Roney S. Coimbra[5], Ana Tereza Ribeiro de Vasconcelos[1*]

*** Correspondence:** Corresponding Author: E-mail: atrv@lncc.br

Genome assembly of *Kqps*142 isolate

The complete genome assembly of KPC-142 isolate, based on 3,804,017 100 bp-reads, was accomplished using a combination of Newbler v 2.6 (Roche Inc.), SPAdes 3.10.0 (Bankevich et al., 2012), GapFiller (Boetzer and Pirovano, 2012) and *phrap/cross_match* (http://www.phrap.org/phredphrapconsed.html) programs. The initial *de novo* assembly using Newbler resulted in 67 contigs >500 bp in length and 36 scaffolds >2,000 bp. The assembly using the SPAdes program resulted in 39 contigs and 25 scaffolds. Since Newbler and SPAdes use different, but complementary characteristics to resolve assemblies, their combined use can greatly improve the resulting assembly. For instance, Newbler usually breaks the consensus sequence in face of repetitive regions in order to avoid possible misassemblies; on the other hand, SPAdes solves small repetitive regions by extending the consensus. We used these different aspects in order to obtain a hybrid assembly that was result of merging both outputs, thus attaining an assembly with a reduced number of scaffolds. The *cross_match* tool was used to align the contigs/scaffolds of each assembly against the other.

To perform gap closure, we initially used *phrap* with consensus sequences from the SPAdes assembly to close the gaps between contigs and scaffolds generated by the Newbler assembly. Then, these consensus sequences were aligned against a database of complete genomes from the *Klebsiella* genus to determine the orientation and order of the contigs and scaffolds (Supplementary Figure S2). The remaining gaps, intra- and inter-scaffolds, identified and not closed by the contigs from the SPAdes assembly were filled by individual assemblies of the reads falling in both termini of a given gap (Supplementary Figure S2). This was accomplished by selecting reads that formed the end of contigs adjacent to each gap stretches. Those reads were assembled separately with Newbler. Contiguous sequences generated by this approach that were able to complete the gap and anchor on the two adjacent contigs were added to the sequence, thus closing the gap. As a last step of gap closure, the 8 remaining gaps (corresponding to rRNA regions), were closed using the GapFiller program.

Figure S2. An illustration of the assembly and gap-closing strategies. Gaps intra- and inter-scaffolds were resolved using an hybrid assembly strategy with the SPAdes and Newbler programs, with local assemblies in gap regions using Newbler.

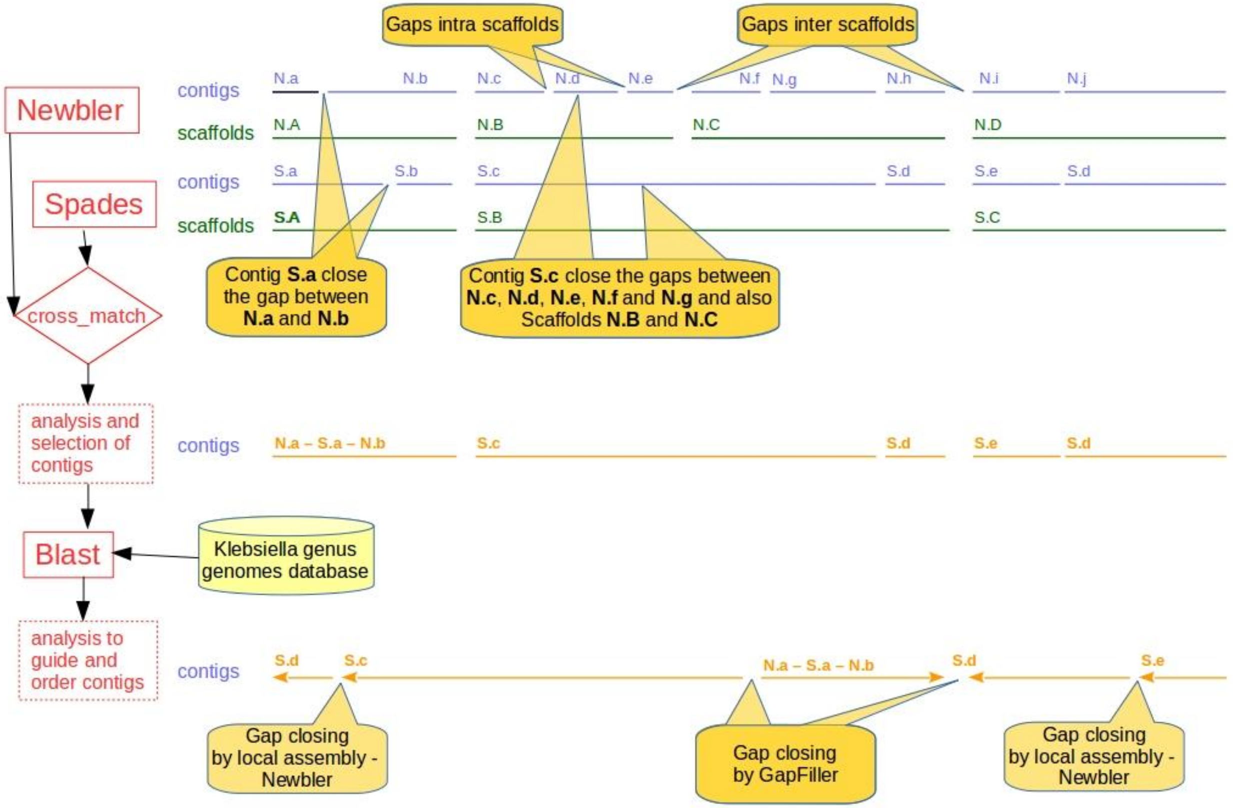

Supplement: Figure S2 — An illustration of the assembly and gap-closing strategies. Gaps intra- and inter-scaffolds were resolved using an hybrid assembly strategy with the SPAdes and Newbler programs, with local assemblies in gap regions using Newbler. [file Image2.pdf]
