# Supplementary figures and images for: Comparative Genomic Analysis of a Clinical Isolate of Klebsiella quasipneumoniae subsp. similipneumoniae, a KPC-2 and OKP-B-6 Beta-Lactamases Producer Harboring Two Drug-Resistance Plasmids from Southeast Brazil
Source: Front Microbiol. 2018 Feb 16;9:220. doi: 10.3389/fmicb.2018.00220 (PMC5820359; doi:10.3389/fmicb.2018.00220)

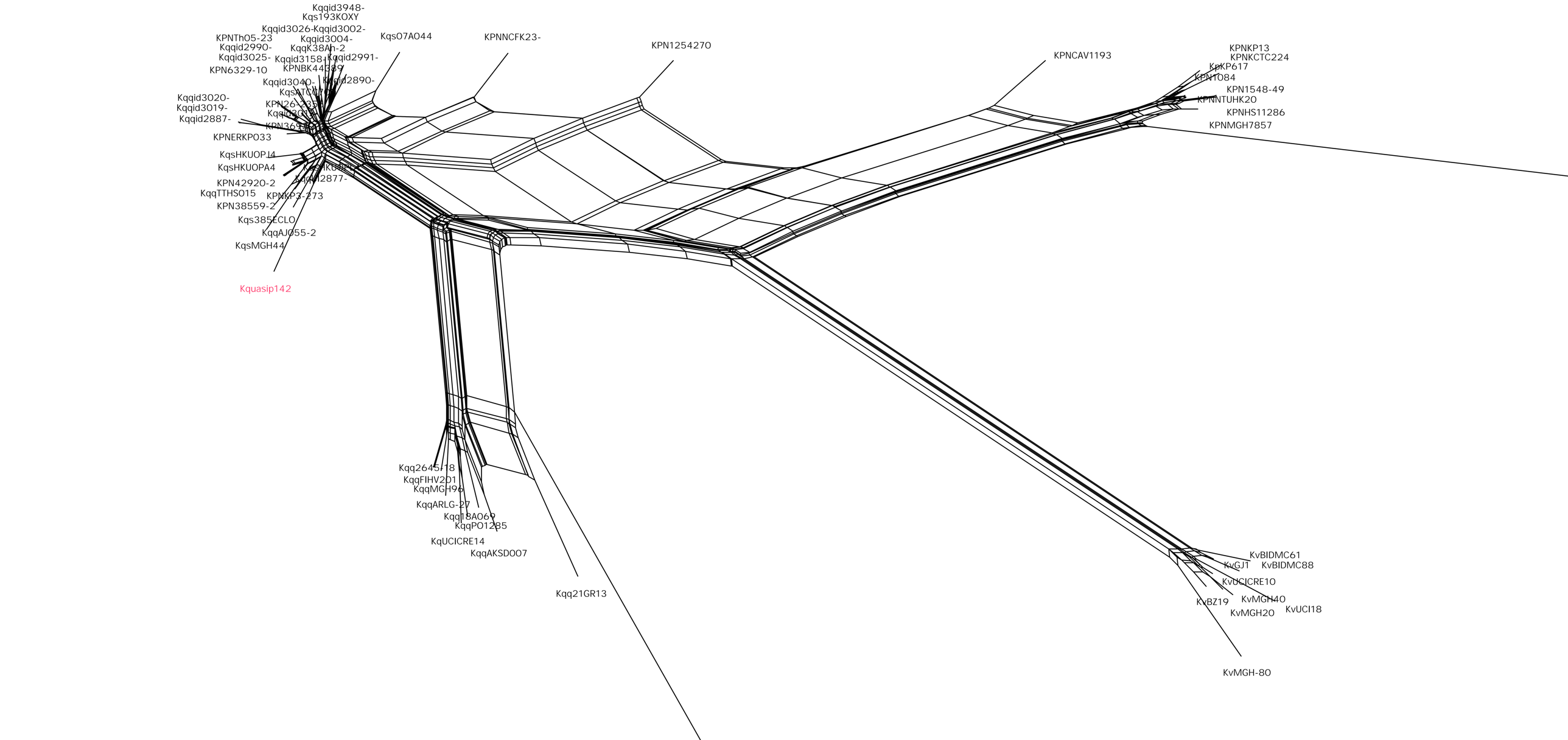

Supplement: Figure S3 — Split decomposition analysis using the seven MLST alleles reveals a network-like structure, suggestive of recombination events. [file Image3.PDF]

*Klebsiella quasipneumoniae* subsp. *similipneumoniae* cell

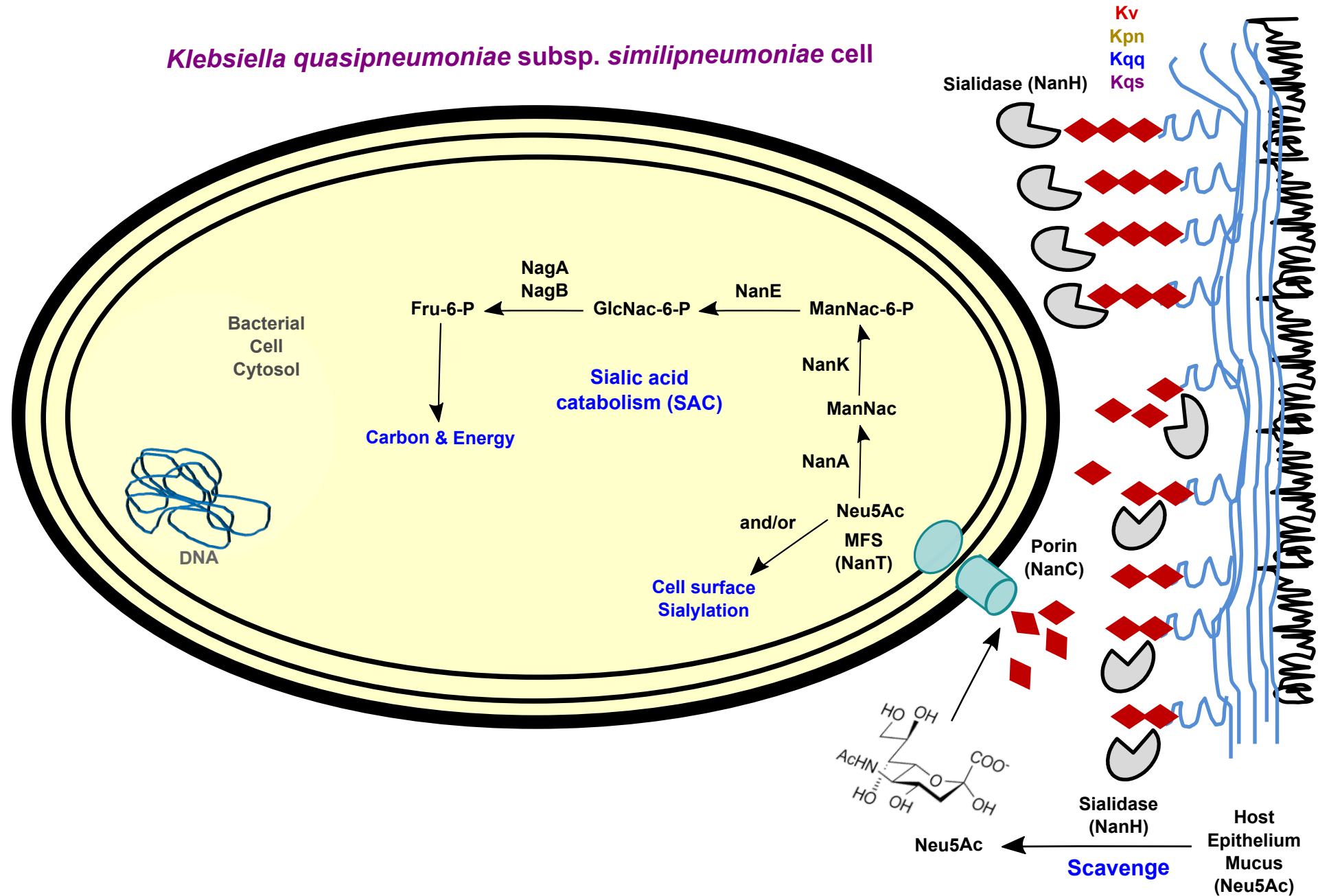

Supplement: Figure S4 — Schematic representation of the utilization of N-acetyl-neuraminic acid (Neu5Ac) in a hypothetical K. quasipneumoniae subsp. similipneumoniae cell. The Kqs cell has potentially the ability to scavenge host Neu5Ac and use it for catabolism and/or sialylation of its cell surface structures, since it carries the gene encoding for a sialidase (nanH), a porin (nanT), sialic acid catabolism (SAC), and cell surface sialylation (Haines-Menges et al., 2015). The genome of Kqq, Kpn, and Kv strains carries nanH encoding for a sialidase and genes encoding for cell surface sialylation, but the genes encoding for SAC are absent, so these strains can potentially use Neu5Ac only for sialylation of their cell surface structures. Kqs, K. quasipneumoniae subsp. similipneumoniae; Kqq, K. quasipneumoniae subsp. quasipneumoniae; Kpn, K. pneumonia; and Kv, K. variicola [file Image4.PDF]
